# Potential Protective Effects of Naloxone in Traumatic Brain Injury Through JAK2/STAT3 Signaling Modulation

**DOI:** 10.3390/life16030480

**Published:** 2026-03-16

**Authors:** Dong Hyuk Youn, Harry Jung, Ji Hyeon Lee, Seongwon Pak, Sung Woo Han, Jong-Tae Kim, Kang Song, Hae Ryong Choi, Gui Seung Han, Young-Suk Kwon, Jeong Jin Park, Jin Pyeong Jeon, Jae Jun Lee, Jong-Kook Rhim

**Affiliations:** 1 Institute of New Frontier Research Team, Hallym University College of Medicine, Chuncheon 24252, Republic of Korea; 2 Department of Biomedical Science, Hallym University, Chuncheon 24252, Republic of Korea; 3 Bio & Health Photonics Research Center Korea Photonics Technology Institute, Cheonan 31035, Republic of Korea; 4 Research & Development Center, Life Genomics Co., Ltd., Suwon 16690, Republic of Korea; 5 Department of Anesthesiology and Pain Medicine, Hallym University College of Medicine, Chuncheon 24253, Republic of Korea; 6 Department of Neurology, Konkuk University Medical Center, Seoul 05030, Republic of Korea; 7 Department of Neurosurgery, Hallym University College of Medicine, Chuncheon 24253, Republic of Korea; 8 Department of Neurosurgery, Jeju National University College of Medicine, Jeju 63241, Republic of Korea

**Keywords:** naloxone, JAK2/STAT3, traumatic brain injury, cognition, neuroinflammation

## Abstract

**Background**: We evaluated the potential neuroprotective effects of naloxone in moderate traumatic brain injury (TBI), focusing on its ability to alleviate neuroinflammation, reduce cognitive impairment, and to influence Janus tyrosine kinase 2 (JAK2)/signal transducer and activator of transcription 3 (STAT3) signaling markers. **Methods**: Male C57BL/6J mice were used to establish an in vivo model of moderate TBI using a stereotaxic impactor. Immediately post-injury, naloxone was administered intraperitoneally (1 mg/kg/day) for 7 days. A total of 72 mice were divided into four groups: Normal, normal with naloxone, TBI, and TBI with naloxone (18 mice in each group). Immunohistochemical analyses and cognitive functions were evaluated across the groups. **Results**: TBI mice treated with naloxone exhibited significantly reduced brain swelling and cortical tissue loss compared to untreated mice. Naloxone reduced Transforming growth factor beta 2 (TGF-β2) and increased interleukin 11 (IL-11) expression in the brain. Additionally, levels of JAK2, STAT3, and B-cell lymphoma 2 (Bcl-2) were significantly elevated following treatment, while expressions of Tumor protein p53 (p53), Caspase 3, Microtubule-associated proteins 1A/1B light chain 3B (LC3B), and Sequestosome 1 (p62) were reduced. Fluorescence intensities of ionized calcium binding adaptor molecule (Iba-1) and dichloro-dihydro-fluorescein diacetate (DCFH-DA) were enhanced, indicating decreased microglial activation and reactive oxygen species (ROS) production due to naloxone treatment. Cognitive function tests revealed improved performance in TBI mice treated with naloxone, demonstrated by decreased alteration rates in the Y-maze test and improved preference index scores in the novel object recognition (NOR) test. **Conclusions**: Naloxone shows potential for neuroprotection and enhanced cognitive performances, which may be associated with modulation of JAK2/STAT3 signaling in a mouse model of moderate TBI.

## 1. Introduction

Traumatic brain injury (TBI) not only causes brain damage through primary mechanical injury but also induces continuous neuroinflammation and histopathological changes through secondary injury [1]. A major contributor to secondary brain injury is an abrupt increase in endogenous opioid peptides following trauma, accompanied by hypoxia, cerebral hypoperfusion, or disruption of the blood–brain barrier (BBB) [2,3]. Endogenous neuropeptides such as β-endorphin significantly increase in the cerebrospinal fluid (CSF) within 24 h after trauma [3]. Inappropriate increases in opioids activate protein kinase C (PKC), mitogen-activated protein kinases (MAP), and brain-derived neurotrophic factor (BDNF), thereby exacerbating oxidative stress, apoptotic cell death, and neuronal inflammation in the damaged brain [4,5]. In this context, Naloxone, a well-known opioid antagonist, has been extensively investigated as a treatment for neurological and cognitive improvement after brain injury. Nevertheless, the efficacy of naloxone for TBI has shown mixed results in vivo studies and clinical trials [2,6]. The main reason for the differences in these controversial study outcomes may be attributed to variations in TBI severity and the adequacy of naloxone’s therapeutic mechanism. Severe brain injuries, defined as a Glasgow coma scale (GCS) of less than 8 on admission, are strong predictors of poor outcome [7]. Additionally, opioid may reduce neuroinflammation and stabilize the BBB, thereby improving neurobehavioral functions after TBI [7]. Therefore, unlike previous studies, to understand the therapeutic effect of naloxone on post-traumatic injury, it may be more effective to observe the effects of naloxone in conditions with less severe TBI, such as moderate TBI, while focusing on other therapeutic mechanisms rather than opioid-related effects.

Mild TBI often presents with transient symptoms and minimal histological damage, making it difficult to detect consistent therapeutic effects in preclinical models. In contrast, severe TBI is frequently associated with irreversible damage, extensive tissue loss, and a narrow therapeutic window, which may obscure or limit the efficacy of pharmacological interventions such as naloxone. Moderate TBI, on the other hand, produces reproducible functional deficits and histopathological alterations while preserving sufficient tissue viability for meaningful therapeutic modulation. Therefore, moderate TBI represents a clinically and experimentally relevant model that allows for the investigation of neuroprotective mechanisms—such as Janus tyrosine kinase 2 (JAK2)/signal transducer and activator of transcription 3 (STAT3) signaling enhancement—under conditions that are both pathologically significant and responsive to intervention.

The JAK2/STAT3 pathway is known to regulate cell proliferation and survival, as well as angiogenesis after TBI [8]. TBI significantly induces JAK2 phosphorylation and STAT3 gene expression in the injured tissues within 3 h after insult [9,10]. Partial inhibition of JAK2 and STAT3 phosphorylation after TBI worsens neurological outcomes. Conversely, activation of JAK2 and STAT3 phosphorylation contributes to decreased apoptosis in the injured cortex [8,10]. Thus, enhancing the JAK2-STAT3 signaling pathway can be a potential therapeutic target after TBI. The JAK2/STAT3 signaling pathway exerts neuroprotective effects through multiple mechanisms: it promotes neuronal survival by upregulating anti-apoptotic proteins such as B-cell lymphoma (Bcl-2) and B-cell lymphoma-extra large (Bcl-xL), modulates microglial and astrocytic responses to reduce excessive inflammation, and facilitates tissue regeneration by enhancing angiogenesis and expression of neurotrophic factors. After TBI, this pathway is rapidly activated as an endogenous protective response against oxidative stress and injury-induced apoptosis.

Naloxone is primarily known as an opioid receptor antagonist, but accumulating evidence suggests it may exert additional effects beyond opioid blockade. In the context of TBI, endogenous opioid peptides are rapidly elevated and contribute to secondary injury via multiple signaling pathways, including those regulating inflammation and apoptosis. Naloxone’s antagonism of opioid receptors may indirectly modulate intracellular signaling cascades such as JAK2/STAT3 by reducing opioid-induced activation of kinases and inflammatory mediators. Additionally, some studies suggest that naloxone can modulate microglial activity and cytokine production independently of opioid receptor antagonism, potentially influencing the JAK2/STAT3 pathway through effects on neuroinflammation and cell survival [11,12]. Moreover, sustained activation of JAK2/STAT3 has been shown to improve functional recovery by regulating key cytokines such as IL-10 and IL-11, which play crucial roles in anti-inflammatory signaling and tissue remodeling. Raible et al. [13] reported that phosphorylated STAT3 (pSTAT3) was significantly increased after injury and then returned to baseline levels a week later after TBI. Despite these mechanistic insights, it is still unclear whether naloxone provides meaningful benefits in clinical settings, or what dosage would be required to achieve therapeutic efficacy. To address this gap, we investigated its potential role in neuroprotection and its association with JAK2/STAT3 signaling modulation in a mouse model of moderate TBI, aiming to generate preliminary evidence that may guide future, more comprehensive studies.

## 2. Materials and Methods

### 2.1. In Vivo Modeling

Male C57BL/6J mice (seven to eight weeks of age) were used to generate an in vivo model of moderate TBI, using a stereotaxic impactor (RWD-68099, RWD Life Science Co., Shenzhen, China) consistent with previous studies [14,15,16]. After positioning the mice in a stereotaxic frame under 3% isoflurane anesthesia, the scalp was incised along the midline, and a craniotomy was performed by removing a small portion of the skull to expose the underlying brain tissue. TBI was induced using a craniotomy-based direct impact paradigm. Briefly, a 2 mm blunt tip was aligned perpendicular to the exposed brain surface and positioned over the parietal cortex (M/L = −2.0 mm, A/P = −1.5 mm from bregma; and depth = 2.0 mm) at velocity of 3.5 m/s and a dwell time of 1.0 m/s. Following impact, the removed bone flap was immediately replaced, and the scalp was sutured. Mice were allowed to recover under a warming lamp and were returned to their home cages after regaining spontaneous movement. The severity of TBI was classified based on published criteria. According to Siebold et al. [17] moderate TBI in mice is typically induced with an impact velocity of 4.0–5.0 m/s and a deformation depth of 1.0–1.5 mm. Although our impact velocity (3.5 m/s) is slightly below this range, the deformation depth (2.0 mm) falls at the upper end of moderate TBI or the threshold of severe injury.

Considering consistent cortical damage, measurable brain edema (based on brain water content), and moderate—yet not severe—impairments in cognitive performance (Y-maze and NOR tests), our model is considered representative of moderate TBI. This classification aligns with previously validated protocols.

**Figure 1:**
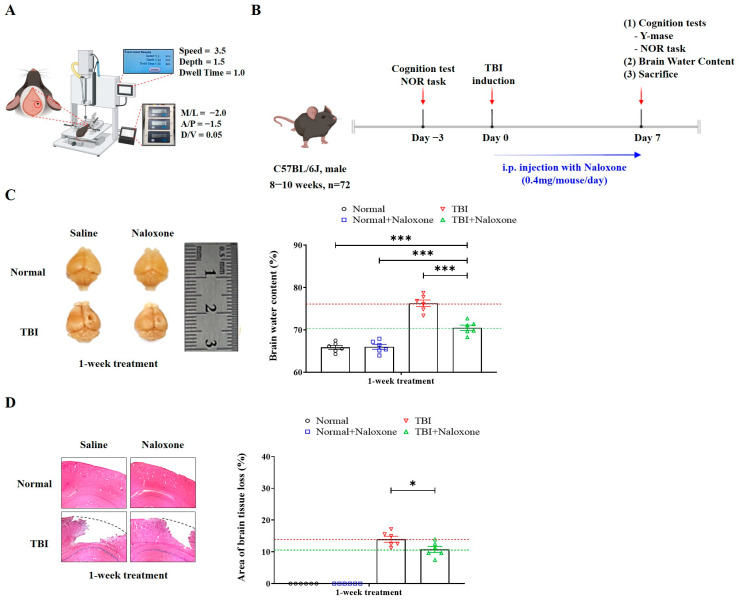
Study design and details of the experiments used in this study. (**A**,**B**) Representative images of cortical injury and quantification of brain water content in response to naloxone treatment. (**C**) Comparisons of H&E-stained histological images and areas of brain tissue loss. (**D**) Scale bar is 200 μm. Error bars indicate SEM. For comparisons involving more than two groups, statistical significance was determined using one-way ANOVA followed by Bonferroni post hoc correction (**C**), whereas Student’s *t*-test was used for two group comparisons (**C**,**D**). * *p* < 0.05, and *** *p* < 0.005.

Naloxone was administered intraperitoneally (IP) starting immediately after TBI induction and continued once daily for 7 consecutive days. The dosage of 1 mg/kg/day was selected based on previous studies reporting its pharmacological efficacy in murine models [18,19]. Specifically, naloxone at this dose has been shown to modulate locomotor behavior [20], attenuate stress-induced neuroimmune changes [21], and exert analgesic effects in mice [22]. All animal procedures were conducted in strict accordance with institutional and national guidelines for the care and use of laboratory animals. The study was approved by the Institutional Animal Care and Use Committee (IACUC) of the Hallym University College of Medicine (Approval No. Hallym 2020-51, Chuncheon-si, Kangwon-do, Republic of Korea,). Mice were housed in a pathogen-free environment with a 12 h light/dark cycle and had free access to food and water. To minimize stress and promote welfare, nesting materials and shelters were provided for environmental enrichment. Animal health and behavior were monitored at least twice daily for trained personnel. Clinical parameters included body weight, posture, grooming, locomotor activity, food/water intake, and sign of distress or neurological dysfunction. During any procedures that could cause pain or discomfort, anesthesia was induced using isoflurane inhalation (1.5~2% in oxygen, via nose cone). Depth of anesthesia was verified by the loss of the pedal withdrawal reflex. Analgesics were not required due to the non-invasive nature of the procedures following TBI induction. Human endpoints were predefined, and mice were to be euthanized if any of the following conditions were observed: (1) 20% body weight loss, (2) inability to access food or water for over 24 h, (3) persistent hunched posture or unkempt fur, (4) unrelieved pain or distress, or (5) severe neurologic dysfunction such as seizures or coma. However, no animals met these criteria, and no animals were found dead during the 7-day experimental period. At the end of the experiment, mice were euthanized using CO_2_ inhalation following cervical dislocation as a secondary physical method, in accordance with IACUC guidelines. Death was verified by cessation of heartbeat, respiration, and loss of reflexes before tissue collection. A total of 72 animals were used and divided into four groups (n = 18 per group): normal, normal with naloxone treatment, TBI, and TBI with naloxone treatment. Analyses of RNA and protein (n = 6), histological staining (n = 6) and brain water content (n = 6) were carried out for each group. No animals were excluded due to adverse health conditions. Each experiment was independently repeated three times. A schematic overview of the experimental timeline is provided in Figure 1B.

### 2.2. Hematoxylin and Eosin Staining of Brain Tissue

Brain tissue samples were collected immediately after sacrifice and promptly fixed in 4% paraformaldehyde for 24 h at room temperature. Then, brain slices (10 µm) were mounted on gelatin-coated coverslips. These sections were rehydrated in distilled water and then immersed in hematoxylin at room temperature for 4 min. After eosin treatment, the sections were dehydrated through a graded ethanol series before immersion in xylene. The hematoxylin and eosin (H&E)-stained sections were visualized using a light microscope.

### 2.3. Brain Water Content, Real-Time Reverse Transcription PCR, and Western Blot

For measuring brain edema, the dry/wet weight method was utilized as follows: % water content = 100 × (wet weight − dry weight)/wet weight [14,15]. To isolate total RNA, brain tissues were processed using the easy-BLUE kit (Invitrogen, Carlsbad, CA, USA). cDNA was synthesized using the Maxime Oligo RT PreMix kit (iNtRON Biotechnology, Seongnam-si, Gyeonggi-do, Republic of Korea). PCR reactions were performed on the Rotor-Gene Q instrument (Qiagen) with the 2× Rotor-Gene SYBR Green PCR Master Mix Qiagen, Germantown, MD, USA). PCR amplification included 36 cycles with denaturation at 94 °C for 15 s, annealing at 55 °C for 30 s, and extension at 70 °C for 30 s [14,15,16]. Glyceraldehyde-3-phosphate dehydrogenase (GAPDH) was used as the normalized control. The primer sequences used for qRT-PCR are listed in the Supplemental Materials and Methods.

For western blot analysis, brain tissues were homogenized with in radio-immunoprecipitation assay (RIPA) lysis buffer supplemented with protease inhibitor K. Then, protein concentrations were measured using the Pierce BCA Protein Assay Kit (Thermo Scientific, Waltham, MA, USA). Equal amounts of protein extracts were separated by SDS-PAGE and transferred to PVDF membranes. Membranes were incubated with the specific primary antibodies, including β-actin (1:1000, #3700, Cell Signaling Technology, Danvers, MA, USA), which was used as the loading control. Band intensities were quantified by densitometry using Image J software (NIH, Bethesda, MD, USA) and normalized β-actin. The primary antibodies used for western blotting are listed in the Supplemental Materials and Methods.

### 2.4. Immunofluorescence Staining

Cryosectioned brain tissues (30 μm thick) were washed thrice in PBS at room temperature to remove the optimal cutting temperature embedding medium. After blocking with 5% normal goat serum and 5% bovine serum albumin for 30 min, the slides were incubated overnight at 4 °C with ionized calcium binding adaptor molecule (Iba-1) antibodies (1:1000; ab178846; Abcam, Cambridge, UK) diluted in antibody diluent solution (E09-500, GBI Labs, Bothell, WA, USA). Following three washes in PBS with 0.05% Tween-20 (PBST), secondary antibodies were applied for 45 min at room temperature. After being washed, the slides were blocked with 5% normal rabbit serum in TPBS and treated with goat anti-rabbit IgG (H + L) antibody (31210, Invitrogen). Secondary antibodies were incubated for 2 h at room temperature. After three in PBST washes, the slides were stained with Alexa 594 goat anti-rabbit secondary antibody (1:1000; 111–586-047; Jackson, West Grove, PA, USA) and dichloro-dihydro-fluorescein diacetate (DCFH-DA). Antibodies are listed in the Supplemental Materials and Methods.

### 2.5. Cognitive Function Test

The Y-maze and the novel object recognition (NOR) tests were conducted to evaluate cognitive impairment referencing previous studies [14,15]. In the Y-maze test, mice were placed at the end of one arm of three-armed Y-shaped maze (length 40 cm, width 10 cm, height 12 cm). Mice were allowed to freely explore the maze for 5 min. The sequence and number of arm entries were recorded, and entry was counted only when all limbs entered the arm. Each arm entry was scored as one point. Spontaneous alternation behavior was calculated using a formula: (number of alternation)/(total number of arms entries) × 100(%) [23]. This test evaluates spatial working memory, a cognitive function primarily dependent on the hippocampus, by assessing the animal’s ability to remember and alternate between previously visited arms. In the NOR test, mice were acclimatized in an open field maze (dimensions: 45 cm length × 45 cm width × 45 cm height) for 10 min per day over two consecutive days. This was followed by object familiarization training conducted twice daily (morning and afternoon) for three consecutive days, with a 4 h interval between sessions. During each session, two identical objects (A1 and A2) were placed in the arena, and mice were allowed to explore freely for 10 min. Seven days after moderate TBI, the test phase was performed using one familiar object (A1) and one novel object (C1). The exploration activity was recorded for 10 min, and preference and was defined as the mouse being within 2 of an object. All behavior was using a video monitoring system, and heat-map analysis was performed using EthoVision software (Noldus Ethovision, Leesburg, VA, USA), where red and blue colors indicated high and low frequency of exploration, respectively [14]. To ensure reproducibility, the maze was cleaned with 70% ethanol between trials, and all tests were conducted by the same trained experimenter in a blinded manner. Each test was conducted by the same trained experimenter in a blinded manner, and all experiments were independently repeated at least three times. Mice were excluded from the analysis if they displayed a strong lateral preference (i.e., spent nearly all exploration time on one object or showed a discrimination index close to 50%) during the training phase. In the test phase, animals that spent less than 60% of their exploration time on the novel object were considered insufficiently trained and were also excluded from the final analysis.

### 2.6. Statistical Analysis

Data were presented as means with standard errors of the mean (SEM). Student’s *t*-test or one-way analysis of variance (ANOVA) with a post hoc Bonferroni correction was performed for all possible pairwise comparisons [14,24,25,26]. Data are presented as mean ± SEM (standard error of the mean), with SEM calculated as the standard deviation divided by the square root of the sample size. Significance levels of <0.05, 0.01, and 0.005 were indicated by *, **, and ***, respectively. All analyses were conducted using GraphPad Prism software (v.8.02; GraphPad Software Inc., San Diego, CA, USA).

## 3. Results

### 3.1. Neuroprotective Effect of Naloxone on Mice with Moderate Traumatic Brain Injury

**Figure 2:**
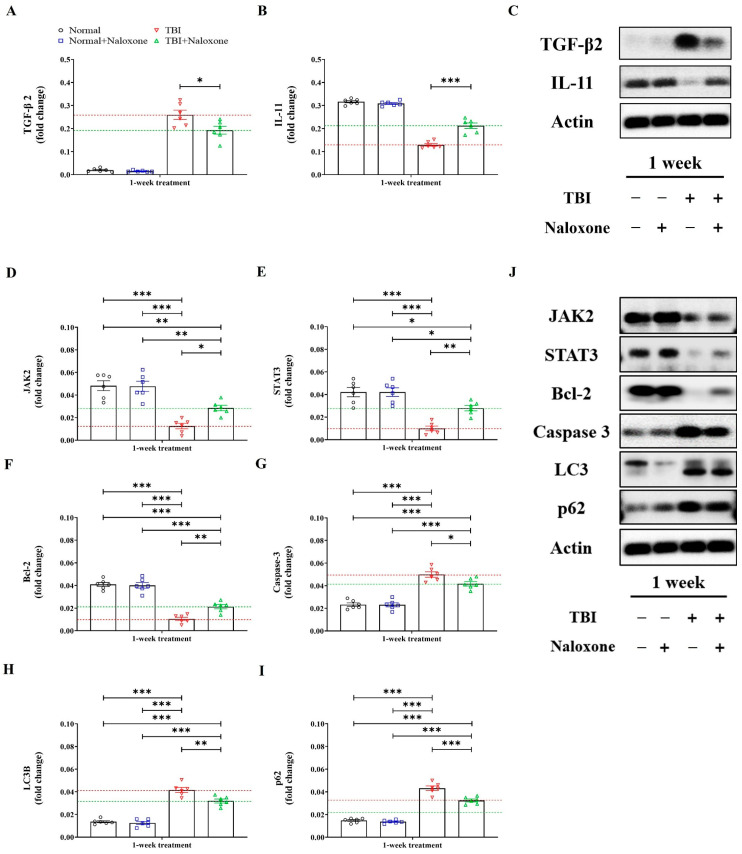
Analysis of differences in mRNA expression and Western blotting for TGF-β2, IL-11 (**A**–**C**), and JAK2/STAT3 (**D**–**J**) signaling-related molecules, including Bcl-2, p53, Caspase-3, LC3B, and p62, following naloxone treatment after moderate TBI. Error bars indicate SEM. For comparisons involving more than two groups, statistical significance was determined using one-way ANOVA followed by Bonferroni post hoc correction (**D**–**J**), whereas Student’s *t*-test was used for two group comparisons (**A**–**J**). * *p* < 0.05, ** *p* < 0.01, and *** *p* < 0.005.

TBI caused cortical damage, tissue loss, and brain swelling. Mice subjected to TBI with naloxone treatment exhibited significantly reduced brain water content and smaller areas of cortical tissue loss compared to those without naloxone (Figure 1C,D). Furthermore, TBI mice treated with naloxone showed significant reductions in TGF-β2 levels and increased IL-11 expressions in the brain, as determined by mRNA analysis and Western blotting (Figure 2A–C).

### 3.2. Expression of JAK2/STAT3 Pathway-Related Molecules

We assessed mRNA and Western blotting for molecules associated with the JAK2/STAT3 signaling pathway. TBI significantly altered the levels of JAK2/STAT3 signaling-associated molecules compared to control groups. Naloxone treatment led to significant increases in the expressions of JAK2, STAT3, and Bcl-2, while it decreased the expressions of p53, Caspase 3, LC3B, and p62 (Figure 2D–J).

**Figure 3:**
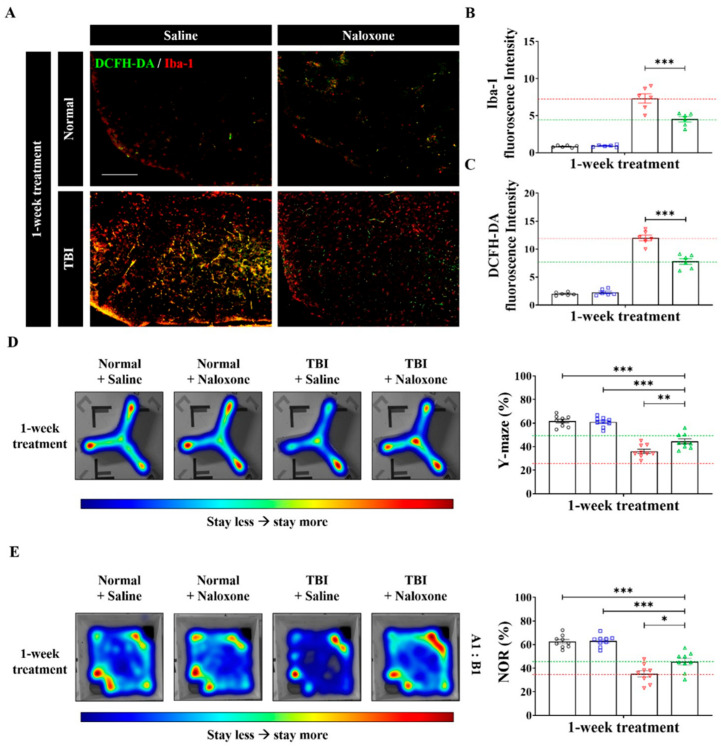
Double-immunofluorescence staining of the injured cortex showing Iba-1 (red) and dichloro-dihydro-fluorescein diacetate (DCFH-DA) staining (green) with quantification of fluorescence intensity across groups with and without naloxone treatment (**A**–**C**). Fluorescence intensity quantification was performed using ZEN 3.4 software, and statistical analysis was conducted using one-way ANOVA followed by Tukey’s post hoc test. (**D**) Cognitive function assessment using the Y-maze test, showing spontaneous alternation performance. (**E**) Novel Object Recognition (NOR) test, showing the preference index score following TBI. In the bar graphs (**B**–**E**), gray indicates Normal + Saline, Blue indicates Normal + Naloxone, red indicates TBI + Saline, and green indicates TBI + Naloxone. Scale bar: 200 μm. For comparisons involving more than two groups, statistical significance was determined using one-way ANOVA followed by Bonferroni post hoc correction (**D**,**E**), whereas Student’s *t*-test was used for two group comparisons (**B**–**E**). * *p* < 0.05, ** *p* < 0.01, and *** *p* < 0.005 indicate statistical significance.

IF staining was conducted to evaluate the expressions of Iba-1, a sensitive marker for microglia activation, and DCFH-DA for ROS (Figure 3A–C). Mice treated with naloxone displayed decreased fluorescence intensities of Iba-1 and DCFH-DA, suggesting reduced microglial activity and ROS production due to naloxone.

### 3.3. Cognitive Function Tests

TBI mice exhibited significantly reduced alteration rates in the Y-maze test and lower preference index scores in the NOR test, indicating cognitive impairment following TBI (Figure 3D,E). TBI mice treated with naloxone demonstrated better performance in cognitive tests compared to untreated TBI mice, exhibiting higher alternation rates in the Y-maze test and improved preference index scores in the NOR test. Detailed protocols for the behavioral tests, including the Y-maze and NOR tests, are provided in the Supplemental Materials and Methods.

## 4. Discussion

**Figure 4:**
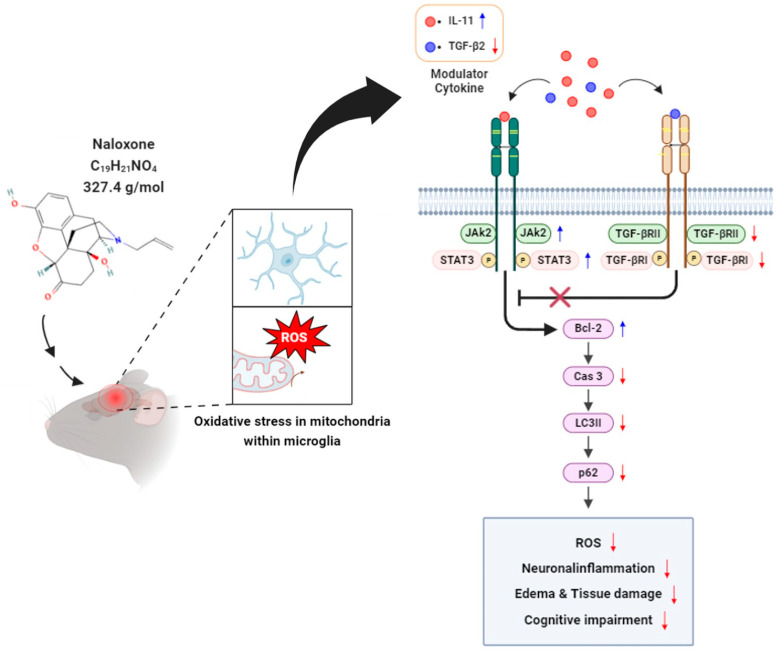
Proposed potential therapeutic effects of naloxone in moderate TBI. Naloxone may help attenuate ROS generation and neuroinflammation after injury, which could contribute to reduced cerebral edema and improved cognitive impairment, potentially associated with modulation of JAK2/STAT3 signaling.

Our findings suggest that naloxone treatment in moderate TBI mice may be associated with modulation of JAK2/STAT3 signaling, accompanied by a decrease in TGF-β2 and an increase in IL-11, which may contribute to reduced neuroinflammation, and brain swelling, and resulting in improved cognitive performance (Figure 4).

Neuroinflammation is a critical secondary process after TBI, largely mediated by microglial activation [27]. While early microglial activation is often neuroprotective, sustained activation exacerbates damage through pro-inflammatory cytokine release. Naloxone, which has a high affinity for mu-opioid receptors (MORs), is widely used clinically to reverse opioid induced respiratory depression [28]. It has a short half-life of approximately 60–120 min [28]. Following observations of elevated endogenous endorphin after TBI [3,4], naloxone has been investigated as a neuroprotective agent that may mitigate secondary injury by modulating the opioid system. Liu et al. [29] reported that naloxone pretreatment reduced nitric oxide (NO) and tumor necrosis factor-α (TNF-α) in LPS-stimulated cultures. Similarly, Tang et al. [30] found that naloxone suppressed microglial activation and Iba-1-expression in both BV-2 cells and mouse brains by regulating ATP-sensitive potassium channels. In our study, we observed a significant reduction in Iba-1 positive microglia following naloxone treatment, indicating suppression of neuroinflammation. Therefore, naloxone likely reduced post-TBI microglia activation and subsequent inflammation-driven damage.

Excessive production of ROS is a hallmark of TBI induced secondary injury. Naloxone has been shown to indirectly modulate ROS by reducing microglial activation and related inflammatory cascades. In our study, naloxone administration led to downregulation of oxidative stress markers and reduced DCFH-DA fluorescence intensity. These findings suggest that naloxone alleviates oxidative damage following TBI. In addition, the activation of the JAK2/STAT3 pathway is known to contribute to the cellular defense against oxidative stress [10,13]. Our findings support this mechanism. Specifically, naloxone enhanced the expression of JAK2 and STAT3 while simultaneously decreasing molecular markers of apoptosis (e.g., p53, Caspase 3) and autophagy (LC3B, p62), reinforcing the notion that this pathway plays a protective role in post-TBI oxidative damage.

TBI often results in long-term cognitive deficits due to neuroinflammatory and oxidative cascades. In our study, naloxone treatment significantly improved performance in both the Y-maze and NOR tests. These behavioral improvements coincided with molecular evidence of reduced inflammation and oxidative stress, suggesting a functional consequence of the observed biochemical effects. Naloxone’s beneficial effects on cognition may also be partially attributed to its modulation of neurotrophic cytokines such as IL-11, as observed in our model. Additionally, previous studies have suggested that sustained JAK2/STAT3 activation contributes to enhanced neuronal survival and functional recovery [8,10,31]. While our findings suggest potential cognitive benefits of naloxone, interpretation of behavioral outcomes requires caution due to methodological considerations. Behavioral testing required predefined exclusion criteria to ensure adequate task engagement; however, these criteria may have introduced selection bias by preferentially retaining animals that explored objects more consistently. In particular, excluding mice based on test-phase novelty exploration thresholds could be viewed as outcome-dependent filtering. Although the criteria were applied uniformly across groups, such exclusions may nonetheless affect the generalizability of the behavioral findings.

Although the therapeutic effect of naloxone has been experimentally substantiated, its clinical efficacy in treating TBI remains controversial. A meta-analysis [2] based on randomized controlled trials revealed that early-stage naloxone treatment resulted in a reduction mortality (OR: 0.51; 95%CI: 0.38–0.67) and severe disability rates (OR: 0.47; 95%CI: 0.30–0.73) 18 months post-injury in patients with severe TBI. However, in mild TBI models, naloxone has been reported to worsen cognitive impairment and depressive-like behaviors in mice, particularly those with high swim stress-induced analgesia [32]. These mixed results suggest that naloxone’s effects may depend on injury severity, timing, and context-specific factors.

TBI is generally categorized into mild, moderate, or severe based on the GCS score. Approximately 80% of TBI cases are considered mild, and most of these patients do not show abnormalities on initial radiologic evaluation. Mild TBI typically resolves with consecutive management without the need for pharmacologic intervention [33,34]. On the other end of the spectrum, severe TBI often involves structural damage such as intracranial hemorrhage with significant midline shift over 5 mm or mass effect, necessitating immediate surgical evacuation. Despite timely surgical intervention, outcomes remain poor due to the persistence of secondary brain injury processes such as cerebral edema, increased intracranial pressure (ICP), and decreased regional cerebral blood flow (rCBF) [35,36].

Naloxone has been reported to mitigate some of these secondary injury mechanisms by improving rCBF and reducing ICP, as shown in experimental model [37]. However, evaluating the effectiveness of naloxone is challenging, given its, limited therapeutic effect in cases of severe TBI with refractory elevated ICP, despite surgery, hyper-osmolar therapy, or sedation. Given these challenges, we focused on moderate TBI. More than 80% of patients with moderate TBI exhibited radiological abnormalities, necessitating interventions to prevent deterioration in the critical care unit [38,39]. Our study demonstrated for the first time that naloxone reduced neuroinflammation and brain swelling, and ameliorated cognitive impairment following moderate TBI.

In this study, we focused on the changes in the JAK2/STAT3 signaling-related molecules following naloxone treatment for moderate TBI. The JAK2 signal transducer and STAT3 activator have shown a protective effect in cardiac ischemic and perfusion injury [40]. Although the mechanisms of cardiac reperfusion injury and TBI damage differ, both conditions share common pathological features such as reactive ROS overproduction and inflammatory cascades. Hence, enhancing JAK2/STAT3 signaling may represent a promising strategy for attenuating secondary injury after TBI. Sudden increases in phosphorylation of JAK2 and STAT3 after trauma gradually decreases, returning to baseline levels within one week [9,10,13]. Zhao et al. [10] reported that traumatized rats treated with recombinant human erythropoietin showed activated JAK2/STAT3 and increased cell survival. Our study also revealed increased expressions of JAK2, STAT3, and Bcl-2, and decreased expressions of TGF-β2, IL-11, p53, Caspase 3, LC3B, and p62 after naloxone treatment in TBI mice.

Although pSTAT3 is the biologically active form responsible for transcriptional regulation, its expression is often transient after brain injury. Therefore, we focused on total STAT3 expression as an indirect marker of the signaling pathway activity. This approach is supported by recent controlled cortical impact (CCI) studies, which reported that both phosphorylated and total STAT3 levels fluctuate depending on injury severity, particularly in neurons and astrocytes [31]. These findings provide a rationale for using total STAT3 expression in our analysis, particularly in models where phosphorylated forms may rapidly return to baseline. Nevertheless, we acknowledge that total STAT3 cannot be interpreted as a direct substitute for pathway activation, which is best reflected by pSTAT3. Instead, total STAT3 should be considered a supportive indicator suggesting involvement of the JAK2/STAT3 signaling, rather than a definitive surrogate for activation. Accordingly, future studies should incorporate acute-phase measurements of both pSTAT3 and total STAT3 to clarify temporal dynamics and mechanistic relevance.

In addition, naloxone treatment attenuated cerebral swelling and neuronal apoptosis, which further supports the hypothesis that JAK2/STAT3 pathway activation contributes to its therapeutic efficacy. Since this pathway is also known to regulate inflammatory mediators, enhance anti-oxidative capacity, and promote tissue repair, sustained activation through naloxone administration may play a multifactorial role in neuroprotection following moderate TBI.

Naloxone exhibits a high affinity for MOR but also binds to kappa-opioid receptors (KOR) [41]. Regarding the association between opioid receptors and JAK2/STAT3 signaling, KOR has been studied more extensively than MOR. Li et al. [42] reported that a KOR agonist significantly reduced ROS production and inflammatory response, along with decreased apoptosis of hippocampal neurons in a model of cardiopulmonary bypass. Conversely, some medications show opposite mechanisms, protecting the myocardium by inhibiting JAK2/STAT3 signaling [40]. This discrepancy may occur due to differences in factors including disease modeling, injury severity, and drug dosage and duration in the experiment [40]. Therefore, additional study is needed to clarify naloxone’s affinity to each opioid receptor and its association with JAK2/STAT3 signaling in neuroprotection against brain injury after trauma.

There are some limitations in our study. First, although we evaluated the neuroprotective effects of naloxone in moderate TBI, the injury severity in our model was more consistent with a moderate-to-severe level rather than a purely moderate severity [14]. Accordingly, the therapeutic effects observed here may not be directly generalizable to mild TBI or to very severe TBI, which may require distinct treatment strategies. Second, the role of opioid receptors in the therapeutic effect through JAK2/STAT3 may vary due to differences in naloxone’s affinity to opioid receptors, requiring further experiments with receptor-specific inhibitors [6]. Third, additional research on the administration route is needed considering clinical applications. After surgery, respiratory depression may occur, leading to aggravation of brain swelling. Due to the short duration of action of modern anesthetics and their reversal agents, the effects of drug-induced respiratory depression are typically not prolonged. Consequently, the administration of naloxone is not necessary solely to counteract respiratory depression [43]. Fourth, cardiovascular effects associated with naloxone should also investigated in TBI conditions. In silico simulations demonstrated naloxone’s ability to prevent cardiac arrest after potent opioid overdose [44]. Clinically, patients show increased endogenous opioids in response to TBI. In particular, in surgical cases, exogenous opioids are often administered during anesthesia for postoperative pain control or sedation. Additionally, naloxone has been shown to increase blood pressure and heart rate through sympathetic nervous system activation, which may be beneficial in reversing TBI-associated hypotension and maintaining cerebral perfusion. Given that endogenous opioid levels are elevated following TBI and exogenous opioids are frequently administered intraoperatively, a better understanding of naloxone’s cardiovascular effects is crucial in clinical settings. Furthermore, naloxone has a relatively short elimination half-life (approximately 60–120 min), and its therapeutic effects typically last for 30 to 90 min. This short duration may lead to underestimation of its long-term efficacy in preclinical TBI models, especially when sustained neuroinflammatory processes are present. In addition, variability in its binding affinity across different opioid receptor subtypes (e.g., mu, kappa, delta) may contribute to inconsistent neuroprotective outcomes. These factors warrant consideration in future studies, ideally through receptor-specific modulators or continuous dosing strategies.

Finally, naloxone and related stereoisomers may act as antagonists of TLR4, thereby suppressing NF-κB signaling and reducing pro-inflammatory cytokine production [45]. Such systemic anti-inflammatory effects could plausibly contribute to attenuation of secondary brain injury following trauma. Given that peripheral inflammation and immune activation are known to exacerbate cerebral edema and after TBI, naloxone’s ability to dampen systemic inflammatory responses may represent an additional mechanism that could underlie its neuroprotective potential following TBI. In addition, future studies should incorporate more specific oxidative stress markers, dynamic autophagy assays (e.g., flux analysis with lysosomal inhibitors), larger behavioral cohorts, as well as to time-course analyses, pathway-specific interventions, drug safety evaluations to better delineate causal interactions and inform clinical applications [46].

## 5. Conclusions

Immediate post-injury administration of naloxone has shown potential to provide neuroprotection and attenuate cognitive impairment in a mouse model of moderate TBI, possibly through modulation of the JAK2/STAT3 signaling pathway. Nevertheless, these observations remain preliminary and are based on limited experimental conditions. Accordingly, further comprehensive studies are necessary to confirm reproducibility, elucidate the underlying mechanisms, and establish the translational relevance of this therapeutic approach.

## Data Availability

The data presented in this study are available from the corresponding author upon reasonable request.

## Supplementary Materials

The following supporting information can be downloaded at: https://www.mdpi.com/article/10.3390/life16030480/s1, Supplemental Materials and Methods: Cognitive function tests. Figure S1: Original unprocessed images of Western blotting used in this study. Table S1: Antibodies used in western blotting and immunofluorescence.
