# Supplementary material for: Potential Protective Effects of Naloxone in Traumatic Brain Injury Through JAK2/STAT3 Signaling Modulation
Source: Life (Basel). 2026 Mar 16;16(3):480. doi: 10.3390/life16030480 (PMC13027423; doi:10.3390/life16030480)
Supplement: Supplementary file 1 [file life-16-00480-s001.zip › Supplemental materials and methods.pdf]

Supplemental Materials and Methods

Cognitive function tests

Cognitive impairment was evaluated through the Y-maze and novel object recognition (NOR) tests. The Y-maze test assessed short-term spatial memory. Mice were placed in a white Y-maze consisting of three arms labeled A, B, and C, each measuring 40 cm in length, 12 cm in height, and 10 cm in width. Starting from the end of one arm, each mouse was allowed to explore the maze freely for 5 minutes. Alternation behavior was identified as successive entries into three different arms. The primary outcome was the spontaneous alternation rate, calculated as the alternation index = (number of alternations / maximum possible alternations) × 100. The NOR test involved habituating mice in an open field arena (46 × 46 × 46 cm) for two consecutive days, 10 minutes each day. Following this, mice were individually exposed to the arena for another two days, with two identical objects (A1 and A2) placed within it. After 24 hours, the mice were tested for 10 minutes in the same arena with one familiar object (A1) and one novel object (B1). Seven days after moderate TBI induction, activity levels were assessed by measuring the distance traveled in the arena during a 10-minute session involving the same object (A1) and a different novel object (C1). Object exploration was defined as any interaction involving sniffing within 1.5 cm of the object, rearing near it, or touching it with the nose. Object preference was recorded when the mouse's nose was within 1.5 cm of the object. All behavioral tests were tracked using a video system and visualized with heat map tracking images (Noldus Ethovision, Leesburg, VA). Blue areas indicated less frequently visited zones, while red areas represented zones visited more often. To ensure the reliability of the findings, all experiments were independently conducted three times, and the data were analyzed in a blinded fashion to minimize bias.

Supplementary Table S1. Antibodies used in western blotting and immunofluorescence

Antibody	Dilution of antibody	Antibody manufacturer	Cat. No.
IL-11	1:1000	Abcam	ab187167
STAT3	1:1000	Cell Signaling Technology	9139S
Caspase-3	1:2000	Cell Signaling Technology	9662S
TGF-β2	1:1000	Abcam	ab13586
Bcl-2	1:1000	Cell Signaling Technology	#3498
LC3B	1:1000	Cell Signaling Technology	2775S
JAK2	1:1000	Cell Signaling Technology	3230S
p62	1:1000	Cell Signaling Technology	#5114
Iba-1	1:1000	Abcam	ab178846
β-Actin	1:1000	Cell Signaling Technology	#5114
Goat anti-rabbit IgG	1:2500	Invitrogen	31210
Alexa 594 goat anti-rabbit IgG	1:250	Invitrogen	A-11012
