# Supplementary figures and images for: Potential Protective Effects of Naloxone in Traumatic Brain Injury Through JAK2/STAT3 Signaling Modulation
Source: Life (Basel). 2026 Mar 16;16(3):480. doi: 10.3390/life16030480 (PMC13027423; doi:10.3390/life16030480)

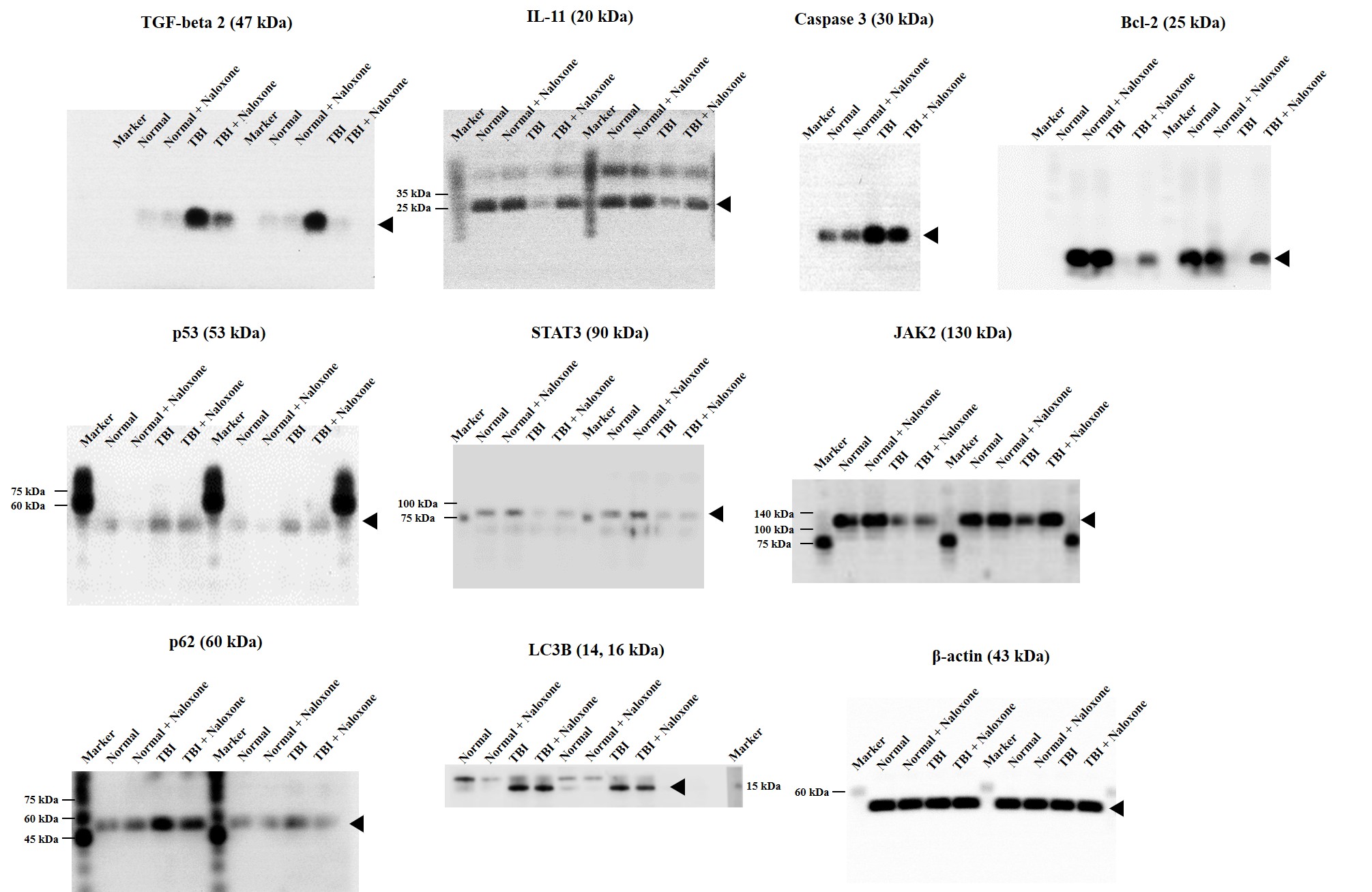

Supplement: Supplementary file 1 [file life-16-00480-s001.zip › Supplementary Figure S1.jpg]

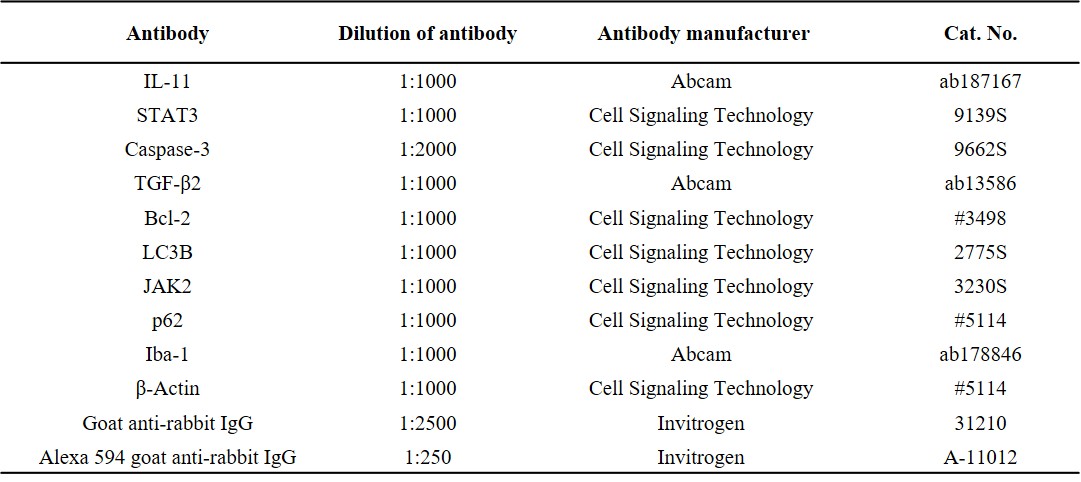

Supplement: Supplementary file 1 [file life-16-00480-s001.zip › Supplementary Table S1.jpg]
